# ANZAED eating disorder credentialed clinician perceptions and experiences of professional development

**DOI:** 10.1186/s40337-025-01307-w

**Published:** 2025-07-16

**Authors:** Janet Conti, Madalyn McCormack, Gabriella Heruc, Katarina Prnjak, Rebecca Barns, Siân A. McLean, Phillipa Hay

**Affiliations:** 1 School of Psychology, Western Sydney University, Penrith, Australia; 2 School of Medicine, Translational Health Research Institute, Western Sydney University, Penrith, Australia; 3 Eating Disorders and Nutrition Research Group, School of Medicine, Translational Health Research Institute, Western Sydney University, Penrith, Australia; 4 Graduate School of Health, University of Technology Sydney, Sydney, Australia; 5 School of Psychology and Public Health, La Trobe University, Melbourne, Australia; 6 Mental Health Services, Campbelltown Hospital, Campbelltown, Australia

**Keywords:** Eating disorders, Credential, Continuing professional development, Qualitative, Supervision

## Abstract

**Background:**

The ANZAED Eating Disorder Credential (the Credential) is the first national and cross discipline program to be developed that specifies the standard of qualifications, knowledge, and ongoing professional development activities needed for health professionals to provide safe and effective treatment of eating and feeding disorders. This study explored clinicians’ experiences and perspectives of the Credential with a particular focus on the ongoing requirements for clinicians to engage in supervision and other eating disorder specific continuing professional development (CPD) activities to maintain their credentialing.

**Methods:**

Participants were 28 ANZAED Credentialed Eating Disorder Clinicians who participated in a semi-structured interview after completion of an online self-report survey. The interview and survey explored their views on the credentialing of clinicians, motivations to become credentialed, and experiences and perceptions of the CPD requirements. Analysis involved descriptive statistics of survey responses and an inductive thematic analysis of interview transcripts.

**Results:**

The thematic analysis generated two main themes with three subthemes for each. The first theme explored the clinicians’ priorities for CPD including supervisor expertise and competence, the supervisory relationship, and accessing and meeting the requirements for CPD. The second theme was on the clinicians’ experiences of CPD focusing specifically on the development of knowledge and competency, support and reflective practice, and the supervisory context. Cutting across these two main themes were the clinicians’ level of experience and clinical practice in eating disorders, and enablers and barriers to CPD.

**Conclusions:**

Credentialed clinicians found the CPD requirements of the Credential, including supervision and other CPD activities, to be valuable. However, many questioned the sustainability of maintaining the Credential, perceiving the requirements as challenging to meet due to time, cost, or access. There is a need for consideration of how to embed greater flexibility in the CPD requirements to account for a clinician’s level of experience, clinicians’ developmental needs, and professional circumstances.

**Supplementary Information:**

The online version contains supplementary material available at 10.1186/s40337-025-01307-w.

## Abstract (plain language summary)

Continuing professional development (CPD), includes both supervision and other CPD activities such as training, workshops, conferences, reading books, and journal articles. CPD supports clinicians to maintain and improve their knowledge, skills and clinical practice when working with people living with an eating disorder (ED). Until recently, there was no clear standard for the qualifications, knowledge and CPD requirements for health professionals to ensure the safe and effective treatment of eating and feeding disorders. The introduction of the ANZAED Eating Disorder Credential (the Credential) aimed to address this issue. The current study explores ANZAED credentialed clinicians’ experiences and perspectives of CPD leading up to and during the period of their credentialing. The credentialed clinicians highlighted the value of the ongoing requirements of the Credential. However, many clinicians discussed concerns around the need for greater flexibility to account for the individual needs of each clinician and queried the sustainability of these requirements due to challenges associated with time, cost, and access.

**Supplementary Information:**

The online version contains supplementary material available at 10.1186/s40337-025-01307-w.

## Background

Continuing professional development (CPD), including supervision and other CPD activities, aims to scaffold clinicians from theory to clinical practice, as they navigate the specific and complex challenges faced by people living with an eating disorder (ED) [1], including medical safety [2], and harm minimisation [3]. There is limited research into CPD experiences of ED clinicians, including what they perceive to be helpful in their clinical work. This paper explores the CPD experiences of ANZAED (Australian and New Zealand Academy for Eating Disorders) credentialed clinicians leading up to, and during the period of their credentialing as endorsed specialist ED clinicians, and their perceptions of the CPD requirements to maintain the Credential. The term continuing professional development (CPD), and ongoing professional development (OPD) are used interchangeably in this paper and includes supervision and other CPD activities (e.g., training, workshops, conferences, reading books and journal articles etc.). The ANZAED credentialing system uses the term ongoing professional development.

While it is clear that CPD is imperative to the delivery of safe and effective ED treatment [4], until recently the minimum requirements for CPD to optimise safe professional practice were unspecified. Alongside the National Eating Disorders Collaboration (NEDC) Workforce Core Competencies [5] and the ANZAED Clinical Practice and Training Standards [6] the introduction of the ANZAED Eating Disorder Credential (the Credential) provides a minimum standard for eligible clinicians. The Credential is, to our knowledge, the first national and cross discipline program to be developed that specifies the qualifications, knowledge, and ongoing professional development activities required for health professionals to provide evidence-based treatment of eating and feeding disorders in Australia [7, 8]. While the Credential is anticipated to improve delivery of care, it is currently voluntary. There is no specific requirement from certifying boards or similar for clinicians providing ED treatment to be credentialed. Furthermore, it is not linked to any financial incentives from government or private health care providers e.g., a higher salary or government-funded service rebates. The Credential has the potential to contribute to the capability of the workforce and increase transparency for consumers and referrers regarding clinicians who meet the minimum standards of skills and knowledge. It also allows clinicians to have their skills and experience publicly recognised [8]. However, the introduction of a credentialing system may risk creating an additional barrier to accessing care or deter clinicians from working in the area of EDs as they would need to undergo additional training and professional development. Thus, it is important to investigate the feasibility and acceptability of the Credential. 

When the Credential was introduced in November 2021, professionals were able to become credentialed under a “sunset clause” whereby their prior experience and training were presented and evaluated without the need to provide documentary evidence of participating in training. This was time limited, ending in June 2022, and is no longer available. This pathway to becoming credentialed has been superseded by the only current pathway that requires professionals to meet the set criteria of prior clinical experience (a minimum of 2 years) and have undertaken introductory training and treatment provision training in an NEDC approved course [9]. An additional pathway was temporarily (from March 2022 until June 2023) offered and comprised a government-funded professional development package delivered by NEDC including free training and/or supervision, referred to here as the “NEDC packages”. These packages aimed to enable clinicians to meet eligibility criteria and incentivise them to become credentialed. These were developed for less experienced clinicians (receiving both training and supervision) and more experienced clinicians (receiving supervision only). The NEDC packages were completed by 896 mental health professionals and dietitians, of whom 524 have gone on to become Credentialed (38.3% of all credentialed clinicians) as of 30 June 2023 [10].

Once Credentialed, clinicians are required to participate in a minimum of 15 h of CPD per year that is relevant to EDs, including 6 h of supervision relevant to eating disorders, and at least 3 h of individual supervision to maintain their Credential. Clinicians are required to keep a record of their CPD activities, which may be subject to auditing [9]. The development of the Credential’s CPD requirements were based on the views of over 650 individual stakeholders gathered through the broad credentialing development consultation process [11]. The CPD requirements for the Credential overlap to varying degrees with the regulatory bodies’ requirements for registration across the different professions that are currently eligible for the Credential in Australia (See Additional File 1).

The majority of research into CPD programs for EDs focuses on the supervision component and is predominantly from the field of psychology. This research on ED supervision emphasises the imperative to address medical instability [12], suicide risk [13], and comorbidity. Research has also focused on the role of the supervisor in supporting clinicians in the development of skills [14] and adherence to manualised evidence-based ED treatment [15]. Deviation from delivery of research evidence-based interventions has been termed ‘therapist drift’ where clinicians are positioned as ‘fail(ing) to deliver the optimum evidence-based treatment’. Within this framework, the task of the supervisor is to remediate ‘therapist drift’ to support flexible implementation of manualised ED treatments through keeping the clinician ‘on track’ in their implementation of these ED interventions [16]. Such approaches recommend that the role of supervisors is to address clinician thoughts, feelings and other characteristics that get in the way of applying empirically supported therapies [17]. On the other hand, it has also been found that supervision risks clinician burnout [18] if it is predominantly focused on individual case formulations with rigid adherence to manualised treatments where ‘any therapeutic departures are scrutinized and interrogated’ by supervisors. 

The literature on ED specific supervision for other professions is limited and concentrates primarily on peer group supervision models. For example, an evaluation of the online Queensland Eating Disorder Service (QuEDS) facilitated peer supervision program for dietitians found that dietitian supervisors perceived that the program increased their capacity to provide supervision, and dietitians perceived that this supervision improved their confidence and clinical practice [19]. Other supervision literature has focused on the role of supervisors in attending to the professional functioning and wellbeing of the clinician to prevent clinician burnout that has been found to be higher in inexperienced ED clinicians [20, 21]. Contributors to burnout have included clinician challenges that arise in contexts of increased ED symptom severity, the therapeutic relationship, time pressures, and financial demands [22]. In response to this, some supervision approaches have proposed that supervisors draw on specific therapeutic interventions, such as Dialectical Behaviour Therapy (DBT), to ‘manage supervisee stressors and challenges’ in their work [23]. Others have recommended that supervision also addresses clinician countertransference reactions [24], including parallel process [25], that have been found to be influenced by clinician gender and patient characteristics, and that have potential to impact the therapeutic alliance and outcomes for those living with an ED. Further to this, ethical dilemmas have also been raised as to when and how a supervisor might raise concerns with a clinician who is also living with an ED [26].

Other than supervision, research into clinicians’ experiences of other ED specific CPD is limited and generally focuses on the provision of ED specific training. Clinicians across the various health professions who provide care for EDs perceive that they have received minimal or insufficient training in EDs both during their tertiary education and on an ongoing basis [27–29], often citing access and finances as barriers. While ED specific training is only one component of CPD, research has demonstrated that it enhances the competence, confidence, and willingness of health professionals to provide treatment to individuals with EDs [27].

Given the diverse views of the what, when, and how of clinical supervision that is optimal for ED clinicians and the paucity of research on CPD training in EDs, there is a need for research to further explore and understand clinician experiences of these when working with different groups of people with a lived ED experience.

With the need for Credentialed Eating Disorder Clinicians to engage in CPD, this study sought to develop a further understanding into credentialed clinicians’ perceptions and experiences of CPD requirements for the Credential and their experiences of CPD, in their work as ED clinicians. This study explores the clinicians’ experiences of supervision and other CPD relevant to EDs, to inform optimal approaches for clinicians who specialise in the treatment of EDs. Through analysing clinician experiences and perspectives, this study aims to inform the ongoing development of the Credential and other training and professional recognition programs, with a specific focus on the requirements for clinicians to engage in ongoing professional development to maintain their credentialing.

## Methods

### Design

This study was a mixed methods design that included descriptive statistics from closed ended survey questions, and an inductive thematic analysis of semi-structured interview transcripts with credentialed clinicians that focused on their perceptions and experiences of CPD as part of the Credential.

### Participants

Participants were 28 ANZAED Credentialed Eating Disorder Clinicians who [1] completed a self-report survey about their experiences and perceptions of the Credential and CPD requirements; and [2] volunteered to participate in a semi-structured interview after indicating interest on completion of the survey. Most of the clinicians (n = 25, 89.3%) identified as female and the mean age was 41.9 (SD = 13.9). The most common ethnicity reported by 11 (39.3%) participants was Oceanian (e.g. Australian, New Zealander, Polynesian). The second most common was European (e.g. British, Irish, Western European, Northern European) or “Oceanian and European” (n = 10, 35.7%). The other remaining 7 (25%) participants were either North American, Asian, African, mixed, or preferred not to say. Table 1 provides further details on the characteristics of the credentialed clinicians who participated in this study.

**Table 1:** Characteristics of Credentialed Eating Disorder Clinicians (n = 28)

Clinician characteristics	Mean ± SD
Age (years)	41.9 (13.9)
Clinician years of experience	12.6 (10.7)

	n (%)
Years of ED specific experience	
< 5 years of experience	14 (50%)
5 + years of experience	14 (50%)
Identified gender	
Female	25 (89.3%)
Profession	
Psychologist	9 (32.1%)
Dietitian	10 (35.7%)
Other mental health professional^*^	9 (32.1%)
Clinical area of work^#^	
Anorexia Nervosa	23 (82.1%)
Bulimia Nervosa	21 (75%)
Binge Eating Disorder	23 (82.1%)
Atypical Anorexia Nervosa	19 (67.9%)
Practice setting	
Private	11 (39.3%)
Public	6 (21.4%)
Private & public	11 (39.3%)
Location of practice	
Metropolitan	18 (64.3%)
Regional, rural, or mixed	10 (35.8%)
Treatment format	
In person	4 (14.3%)
Telehealth	4 (14.3%)
Multiple formats	20 (71.4%)
Hours/week of ED work	
< 16 h a week	15 (53.6%)
16 + hours a week	13 (46.4%)
Proportion of clients with an ED	
A few (< 25%)	4 (14.3%)
A substantial number (25–50%)	9 (32.1%)
Many (50–75%)	4 (14.3%)
Almost all/All (75–100%)	11 (39.3%)
Provides clinical supervision	12 (42.9%)
NEDC package recipient	13 (46.4%)

^*^Other mental health professionals included counsellors, mental health nurses, social workers, and a psychiatrist

^#^These are not exclusive categories

### Procedure and materials

This study was approved by the Western Sydney University Human Research Ethics Committee (approval number: H15252).

Participants were identified via the ANZAED Credentialed Eating Disorder Clinician database and were contacted directly using their listed email address (N = 1340) to invite participation in the online Qualtrics survey. This survey collected data from 228 credentialed clinicians and was comprised of a range of closed and open-ended self-report questions first developed by the authors (PH, JC, KP) about clinician characteristics, their current engagement in CPD, including their experiences and perceptions of clinical supervision (including barriers and enablers) and other CPD activities. At the end of the survey, participants were able to leave their email address to indicate their interest in being interviewed further about their experiences. All participants who expressed their interest (n = 57) were contacted, screened for eligibility, and their consent was obtained. Clinicians were included in this study if they met the Credential criteria and were excluded if they declined, were unable to be contacted, had not been practicing since they became Credentialed, or failed to meet the ongoing professional development and supervision requirements of the Credential. Participants were interviewed in order of responding until data sufficiency was reached (n = 28). A small number of clinicians were selected to be interviewed to increase the representation of people from certain disciplines, such as social work. At the point of data sufficiency across professional disciplines, the invitation to participate in the interview was removed from the Qualtrics survey. The participants who took part in both the survey and interview were older (*p* = < 0.043) and saw a larger proportion of clients presenting with Anorexia Nervosa (AN) or Atypical AN (*p* = 0.026) than other ED presentations in comparison to those participants who took part in the survey only. However, there were no significant differences in their gender, profession, practice setting and location, treatment format, years of experience working generally and with EDs, number of hours spent with ED clients per week, and supervisory experience (see Additional File 2).

Semi-structured one-on-one interviews that ranged between 45 and 60 min were carried out by three researchers (KP, RB, NO). Interviews focused on clinician views on the credentialing of clinicians, motivations to become credentialed, clinicians’ expectations and priorities for supervision, and experiences of supervision. For clinicians who were supervisors, open ended questions also enquired into their perspectives on the skills and challenges of providing supervision to clinicians who specialise in the treatment of EDs (further details on the interview questions can be found in Additional File 3).

Interviews were audio recorded, transcribed verbatim, and de-identified with a participant number. Transcripts were then given to participants to “member check” for accuracy and the removal of further identifying information for confidentiality.

### Analysis

Analysis of clinicians’ experiences and perceptions of CPD was undertaken through descriptive statistics of survey responses and an inductive thematic analysis that generated themes from transcripts of the semi-structured interviews [30]. An inductive approach to the thematic analysis was appropriate given that there is little known about ED clinicians’ experiences and perspectives of credentialing. Themes were constructed through the explicit language used by participants, and an analysis of implicit meanings in their narratives, as language in this instance is assumed to construct a version of the participants’ experiences and meaning-making processes [31]. Two authors (JC & MM) familiarised themselves with the data by reading and re-reading transcripts, coding meaningful units of raw data and sorting these into large file documents. Within these large file documents, initial codes were then collapsed into overarching codes followed by memo writing to develop emergent themes [32]. These emergent themes were discussed with an additional author (PH) to refine overarching themes and a thematic map that addressed the research questions. For each theme and subtheme vivid exemplar quotes were selected and followed by an in depth analysis of these as outlined in this paper (JC, MM, PH) Exemplar data extracts from the clinicians semi structured interviews can be found in Additional File 4.

Participating clinicians were invited to “member check” their interview transcripts for the purposes of validity and to align analysis of specific quotes with participant feedback. Two clinicians provided minor feedback that was mainly grammatical and did not change the meaning of their transcript.

## Results

Analysis of clinicians’ experiences of CPD comprised descriptive survey data, and thematic analysis of interview transcripts. The thematic analysis of clinician experiences and perceptions of CPD to maintain the Credential clustered around three main themes with embedded subthemes (Box 1).

**Box 1:**
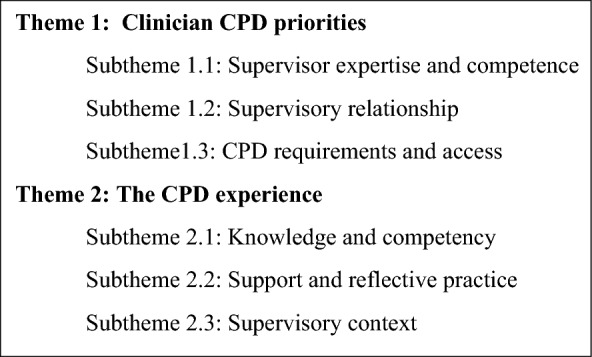
Clinician experiences and perceptions of CPD to maintain the Credential

**Fig. 1:**
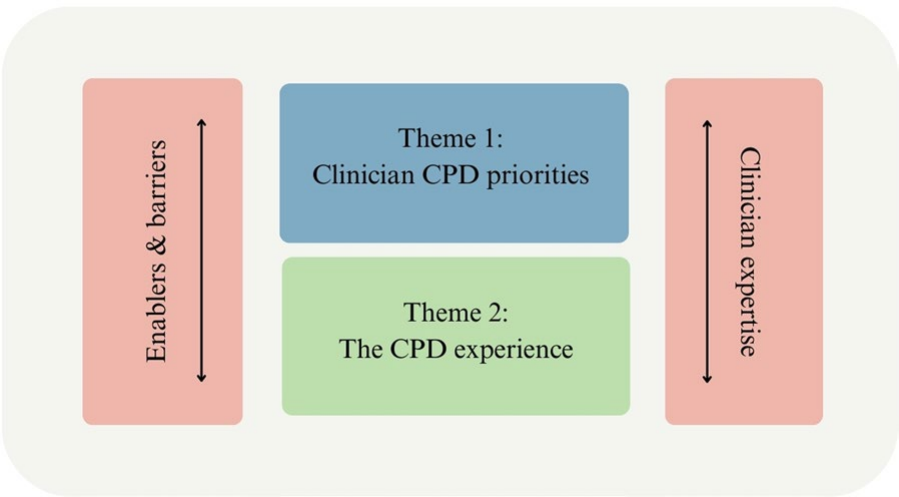
Clinicians’ CPD priorities and experiences: Cross cutting themes

The nature of clinician talk was also shaped by two cross cutting themes as illustrated in Fig. 1 that were (1) Clinicians’ experience and clinical practice in EDs; and (2) Enablers and barriers to CPD. These cross-cutting themes were additional issues and areas that intersected with the two main themes. Clinician CPD priorities and experiences were therefore shaped by perceived enablers and barriers to CPD as well as by the level of expertise of clinicians.

### Theme 1: clinician CPD priorities

Clinicians CPD priorities focused primarily on supervision with less consideration given to other CPD activities such as training, workshops, conferences etc. The majority of clinicians reported both group and individual supervision experiences, with half describing experiences of supervision in interdisciplinary and/or peer contexts. Clinician priorities included supervision with an experienced clinician, and the ongoing supervisor requirements to maintain the Credential were perceived to be reasonable. Although around 50% of the clinicians felt well equipped to provide supervision, the majority identified a need for more specific ED supervisor training and guidelines. Table 2 summarises clinicians views on their preferences and perceived barriers and enablers of CPD.

**Table 2:** Clinician (n = 28) views on their preferences and perceived barriers and enablers of Continuing Professional Development (CPD)

Aspect of CPD	n (%)
**Priorities and preferences:**	
Format of supervision^*^	
Combination of individual & group	22 (78.6%)
Interdisciplinary	15 (53.6%)
Peer	13 (46.4%)
Preference for supervisor years of experience (n = 26)^#^	
< 5 years of experience	7 (26.9%)
5+ years of experience	19 (73%)
**Perceived barriers and enablers:**	
Barriers to supervision^*^ (n = 24)^#^	
Time	20 (83.3%)
Cost	14 (58.3%)
Supervisor availability	8 (33.3%)
Enablers to supervision^*^ (n = 26)^#^	
Regular supervision	22 (84.6%)
Shared understanding	14 (53.8%)
Similar characteristics, beliefs & values	14 (53.9%)
Trust	22 (84.6%)
CPD supervision requirements of Credential are reasonable (n = 26)^#^	21 (80.8%)
Other CPD requirements of Credential are reasonable	26 (92.9%)
Clinicians feel well equipped to provide ED supervision	14 (50%)
The following support clinicians in ED supervision:	
ED specific training	24 (85.7%)
ED supervisor guidelines	21 (75%)

^*^These are not exclusive categories

^#^ Note for some survey responses the number is less than 28. These survey items were not set up to force a response and some participants chose not to respond

The clinician interviews expanded upon the importance of goodness of fit or ‘match’ (C8, Dietitian; C12, Mental Health (MH) Nurse; C26, Psychologist) when finding a supervisor. A substantive proportion of interviewed clinicians preferred supervision with a supervisor who practiced and/or had experience in the same model of ED care. Clinicians also valued a supervisor who aligned with their professional needs, practice context, with shared principles and expectations for supervision, similar work experience, and/or areas of speciality (e.g., bariatrics), and for some, autonomy in choosing their supervisor. Clinicians with access to supervision external to their organisation experienced this as beneficial; for example, in reducing bias when working with individuals known to the organisation (C4, Dietitian), creating a safe space to talk about challenges (C9, Dietitian), and to name difficult work dynamics referred to by C13 (MH Nurse) as unspoken (the *‘elephant in the room’*) in a workplace.

Clinicians had diverse perspectives on the profession of their supervisor. While some believed that it was useful to collaborate with other professions in order to stretch their perspective, prevent clinician siloing (*‘go(ing)down their silos’* -C22, Dietitian) and develop their skills and knowledge, others argued that supervision should be conducted with a supervisor from the same profession. This was particularly the case for psychologists who have regulatory body requirements around supervision (see Additional File 1) and dietitians who noted elements of their practice that would need a profession specific supervisor (e.g., *‘setting expected body weights’*- C4, Dietitian).

Clinician priorities for CPD, including supervision, clustered around three subthemes. These were supervisor expertise and competence (subtheme 1.1), the supervisory relationship (subtheme 1.2), and CPD requirements and access (subtheme 1.3).

#### Subtheme 1.1: supervisor expertise and competence

Consistent with the survey findings (Table 2), clinicians, particularly in their early career (< 5 years), valued supervisor expertise with one indicator of this being the preferred years of ED specific experience. Some clinicians emphasised the importance of supervisors having extensive ED specific knowledge, and that this matched with their preferred ways of working. Early career clinicians perceived the key role of supervision as facilitating a practitioner’s development of knowledge and competency in clinical practice. More experienced clinicians (> 5 years) emphasised the importance of supervision when working with individuals who were *‘high risk’* (C9, Dietitian) or *‘serious’* (C28, Social Worker).

More experienced clinicians noted that an experienced clinician does not always translate to an experienced supervisor. For example, in positioning supervision as *‘an art’* C19 (Clinical Psychologist) argued that supervisory practices go well beyond a supervisor’s clinical (or research) skills. Another clinician outlined the importance of a supervisor who was skilled in discerning the moment by moment needs of supervisees: ‘*I like a supervisor who can quite quickly read the room and figure out… where to be directive and where to be more reflective’* (C25, Psychologist).

Clinicians also spoke about the importance of supervisors being skilled in supervisory practices, including setting up supervision appropriately with a contract to ensure a formal supervision relationship with an agenda, clear expectations, and boundaries. Furthermore, several clinicians across the professions (e.g., C19, Clinical Psychologist; C9, Dietitian; C12, MH Nurse) argued that supervisors of credentialed clinicians should be trained in supervision before they facilitate ED specific supervision. One early career dietitian (C5) argued that clinicians do not know what they do not know until they experience supervision with a supervisor trained in supervision: ‘*[Supervisor] has done the training about being a supervisor as well … I guess that some people, probably, who haven’t experienced good clinical technical supervision don’t see the benefit of it.’*

Although clinicians valued supervisors with specific ED experience, there were conflicting thoughts amongst the clinicians about whether they required their supervisor to be an ED credentialed clinician. For example, the Credential was viewed by two clinicians as signposting a supervisor’s expertise and competence, whereas another clinician positioned this as an *‘added bonus’* (C14, Clinical Psychologist) and indicated they would consider supervision with a non-credentialed supervisor.

#### Subtheme 1.2: supervisory relationship

The supervisory relationship was perceived by clinicians as an imperative part of the supervisory process. For example, ‘*I guess, what you would be looking at in any kind of therapist. … an empathic, good listener’* (C1, Clinical Counsellor)*.* This extract highlights the parallel processes of the therapeutic relationship and the supervisory relationship and how this was prioritised when deciding on a supervisor. Aligned with this, was trust in the supervisor that was valued as much as regular supervision (84.6%) (Table 2). Other valued aspects of supervision included shared understanding (53.8%) and similar characteristics, beliefs and cultural values (53.9%). Interview data paralleled these survey findings, where regardless of level of experience, many of the clinicians, implicitly or explicitly endorsed the importance of creating a safe supervisory space through collaboration, rapport, non-judgmental stance, flexibility to meet them where they were at, and a supervisor who was open and approachable. This space facilitated them to genuinely speak about their clinical work, including some of the challenges they faced. Some clinicians spoke about the benefit of their supervisor being external to their workplace as this allowed them to speak about a broader range of issues including workplace pressure, hours, lived experience. This safe space was also talked about as having room for a ‘knowing’ and a ‘not-knowing’ stance, for both clinician and supervisor.

*EXTRACT 1*

*I learned from me, and I learned from them, and we work things out together, which I think makes it easier to have less taboo topics, I guess. … Something that I find really valuable is when they say they don’t know something… (C10, Dietitian)*

This extract highlights how challenging it can be for clinicians to talk about what they ‘*don’t know*’ in their work and the importance of both clinicians and supervisors being comfortable in taking a not knowing stance (or negative capability that ‘centres on suspending judgment about something in order to learn more about it’ [33]) where they engage in a two-way process of learning between clinician and supervisor. C4 (Dietitian) also described how sharing a not knowing stance was helpful in supervision in preserving a sense of professional identity, for example being ‘*OK*’ as a clinician. Implicit in these clinician accounts was the power dynamic in the supervisory relationship and the importance of supervisors prioritising the development of clinician’s professional identity through creating a safe space for supervisees to express vulnerability around challenges in practice. One way this safety was cultivated was around supervisors also being comfortable with a not-knowing stance.

Less experienced clinicians (< 5 years) prioritised certain qualities of their supervisor expressing preference for a supervisor who was warm, compassionate, flexible, empathetic, and patient. This made room for supervision to be a place where clinicians felt ‘*comfortable bringing stuff up*’ (C5, Dietitian), talking about ‘*mistakes*’ (C27, Social Worker), and where ‘*everything was on the table’* (C10, Dietitian). More experienced clinicians (> 5 years) valued a supervisory relationship where there was safety to be challenged (for example, to ‘*provide that like redirection, if it’s needed*’—C11, Dietitian; ‘*more critiquing*’ -C14, Clinical Psychologist; ‘*what might need to be amended and changed’* and *‘when I was working harder than the client*’-C12, MH Nurse) as well being approachable and available for supervision (for example: ‘*be able to set aside the time*’-C23, Dietitian).

Overall, clinicians valued safety in the supervisory relationship. For early career clinicians, this supported the development of knowledge and skills and informed their emerging identity as an ED clinician. For more experienced clinicians, safety in the supervisory relationship supported their ongoing professional identity development, including in the context of being authentic and challenged in their practice.

#### Subtheme 1.3: CPD requirements and access

In the survey, 80.8% and 92.9% of clinicians reported that the ongoing supervision and other ED specific CPD requirements for maintaining the Credential were reasonable, respectively (Table 2). A more nuanced picture was evident in the interviews, where although most early career clinicians described CPD requirements as reasonable and fair, some of the clinicians who held several professional memberships experienced the CPD requirements to be ‘*onerous*’ (C6, Social Worker) and financially burdensome, pointing to consideration of the need for a universal system to log their training to avoid unnecessarily increasing their workload.

There was greater variance in the attitudes towards the other ED specific CPD requirements from more experienced practitioners. While some experienced clinicians perceived the requirements to be reasonable, others failed to see the benefit. Experienced clinicians argued that their level of experience should be taken into consideration and training requirements should be tailored accordingly, including arguments for a ‘*tiered*’ system (C18, Social Worker), or ‘*different levels*’ (C6, Social Worker) of CPD requirements based on clinician years of experience. They also pointed out the importance of CPD including interventions that address the complexities inherent in an ED presentation, such as trauma informed care and interventions, and clinical supervisor training.

Cost, access, and time were identified as primary barriers to completing CPD, particularly for early career clinicians and some professions (e.g. nurses). Access to supervision by an experienced credentialed clinician was identified as particularly challenging for rural and remote practitioners, specialist ED clinicians (e.g. bariatric surgery), and some disciplines, such as social work. Notably, nearly one in five of the clinicians indicated that they had not been able to access supervision that met the requirements for credentialing. On the other hand, access was improved for clinicians through utilising the NEDC package and group (including peer) supervision.

These on the ground challenges in clinicians finding a specialist ED clinician and supervisor were echoed by the following experienced clinical supervisor.

*EXTRACT 2*

*Make sure that you’ve got the trained supervisors to actually do the supervision and that people aren’t just in desperation when their credentialing is going to expire (and) turn to you for supervision (C19, Clinical Psychologist)*

Clinicians also spoke about the need to consider the hours a clinician spent both in the workforce and engaging in ED specific work. For some clinicians, the Credential was perceived as ‘*probably too hard*’ (C7, Dietitian) with one recommendation to pro-rata the CPD requirements with the proportion of a clinicians practice that focuses on ED presentations. This was particularly the case for rural and remote clinicians who had mixed clinical practices. Some of the suggestions made by clinicians to improve access to supervision included supervision groups with open membership, and a service such as a hotline, scholarships or government subsidies, and a library of CPD resources.

Overall, these clinician perspectives call for a clearer understanding of what constitutes CPD relevant to EDs, the recognition that a siloed discipline-specific approach to treating eating disorders and CPD risked impeding a clinicians’ professional development, and the need to tailor CPD by the extent to which a clinician practices with ED presentations. These clinician perspectives highlight the importance of CPD being flexible to grow with the clinician to meet their professional developmental career needs.

### Theme 2: The CPD experience

Most clinicians spoke about the value of engaging in ongoing CPD. Regardless of level of experience, the clinicians perceived supervision as a space to engage in case consultation, stretch perspectives, expand knowledge, resources, and skill sets, feel supported and contained, and engage in reflective practice. However, many clinicians described a lack of inclusion of ED specific training in the university curriculum of many health professions and their subsequent need to take responsibility for their ED specific clinical skills development. For some, the NEDC package alleviated this problem by providing introductory training and supervision about EDs with qualified and experienced clinicians. However, whilst many early career clinicians felt the ongoing requirements for CPD to maintain their credentialing were reasonable, there were concerns around a lack of time, finances, access to training, and support from their workplace. These concerns were echoed by more experienced clinicians who expressed varying attitudes towards the ongoing requirements, with many suggesting that are more tailored approach is needed. Table 3 provides a summary of the clinicians’ endorsement of statements reflecting their perceptions and experiences of CPD. These findings will be discussed in greater detail throughout the thematic analysis below.

**Table 3:** Clinician endorsement of statements regarding aspects of continuing professional development (CPD)

Aspect of clinician CPD experience	n (%)
My experience of supervision was that (n = 26)^#^	
Supervision provides a safe space	26 (100%)
Supervision was tailored to my needs	26 (100%)
Supervision enhanced my.	
Reflective skills	25 (96.2%)
Confidence	25 (96.2%)
Ability to deliver ED-EBI	23 (88.5%)
Ethical thinking & decision making (n = 25)^#^	23 (92%)
My willingness to treat EDs was increased	21 (80.8%)
Supervision relevant to EDs improved my.	
Confidence	22 (78.6%)
Competence	22 (78.6%)
Other ED specific CPD enhanced my.	
Understanding of ED assessment/treatment	27 (96.4%)
Confidence	28 (100%)

CPD = Continuing Professional Development, ED-EBI = Eating Disorder Evidence Based Interventions

^#^Note for some survey responses the number is less than 28. These survey items were not set up to force a response and some participants chose not to respond

#### Subtheme 2.1: knowledge and competency

The clinicians identified the Credential as a way to fill a gap in knowledge and competency due to the paucity of ED specific tertiary education training and argued for the need for EDs to be *‘integrated’* (C16, Psychologist) into university curriculum, including information about the Credential rather than having to ‘*try and figure it out on their own*’ (C13, Nurse). Some interviewees perceived the cost and time required for introductory training and supervision as a barrier to becoming a Credentialed Eating Disorder Clinician, although the NEDC training package was perceived by those who participated as providing improved accessibility. However, one clinician noted that training clinicians at no financial cost also risked this being valued less highly by some clinicians (C9, Dietitian). Another experienced clinician and supervisor expressed concern that the minimum CPD required to become a credentialed clinician may not be adequate for the treatment of particular EDs with complex presentations and treatment needs: ‘*the other issue I have is if you were credentialing people on anorexia nervosa, that would be one thing, because I know that all the cases that you’ve produced, all your knowledge base, but it’s on eight, and I’m not convinced, because I don’t think all eating disorders are equal’* (C19, Clinical Psychologist).

Supervision was also talked about by the clinicians as a key way that they developed and strengthened their knowledge, competency, and confidence in working in the field of EDs. This was evidenced in one clinician’s account of an identity shift from the sense of themselves as an ‘*imposter*’ to a greater sense of ‘*more confidence, competent*’ (C18, Psychologist). This sentiment was supported by the survey responses where clinicians reported engaging in an average of 23 h (SD = 18) of supervision annually and the majority (78.6%) of clinicians surveyed agreed that supervision improves a clinician’s confidence and competence (Table 3).

Supervision was also perceived and experienced as a way for clinicians to maintain *‘model fidelity’* (C18, Psychologist) to specific research evidence-based ED treatment interventions to prevent therapist ‘drift’. For example, ‘*you can get a lot of boundary drift with eating disorders and self-care stuff. I guess, as well that gets checked in, in my supervision’* (C9, Dietitian) and *‘… figure out what you need to do to be more adherent to model’* (C6, Social Worker*).*

On the other hand, more experienced clinicians who were also supervisors highlighted the complexity of the delivery of ED treatment interventions and the importance of supervision as a way to also tailor treatments to the complexities of individual presentations.

*EXTRACTS 3*

*… it’s really important if you’re learning a new type of treatment to be as strict and stick to the fidelity of the original treatment to get that embedded in your mind. It’s a bit like learning to cook, you know, you follow the recipe for a while until you feel confident enough … and bring in a little bit of this, a little bit of that for this person in front of them today … it’s that humans are all different … and that’s why it’s always a therapeutic alliance that comes out as the top in any research. (C24, Psychiatrist)*

*… I can take someone and train them, but you know … if you don’t really understand the individual and the complexities of the individual that you’re providing the treatment to, then the treatment doesn’t work. … For me it’s really getting them to understand the interaction between a technology that they’ve learnt and the recipient of that technology. Just because someone’s an adolescent and has anorexia nervosa it’s not going to mean that family-based treatment is going to work and, in my case, I could argue, it could be harmful. (C19, Clinical Psychologist)*

These experienced clinician and supervisor perspectives highlight the importance of supervision being both a place for checking fidelity to specific evidence-based treatment models and to develop competencies in clinicians to tailor treatments to the needs and preferences of the ‘*person in front of them today*’ (C24, Psychiatrist). Furthermore, supervision was positioned by experienced clinicians as a space to foster inexperienced clinicians to move beyond a “one-sized” fits all approach (C16, Psychologist) that risks failing to be responsive to an individual’s unique needs and cultural context. With one clinician arguing that supervision should invite the clinician to “*[…] understand what they are trying to do rather than checking all the time on whether they’re doing the treatment properly”* (C19, Clinical Psychologist).

Supervision was perceived by other clinicians as a space to seek advice and feedback, and/or a source of external validation that they were ‘*on the right track*’ (C11, Dietitian; C5, Dietitian; C27, Social Worker), share responsibility for safe professional practice (C10, Dietitian; C11, Dietitian), scaffold their training into clinical practice, and increase the sustainability of their work (C17, Psychologist). This included supervisors responding to potential challenges and mistakes in their work in ways that preserved the clinicians sense of professional identity and cultivated a parallel process of safety for both clinicians and the people with a lived ED experience with whom they work. For example: *‘… the psychologist I used to get supervision from, would be really good at identifying when I was working harder than the client, but she had a really good way of … doing it in a way that didn’t feel like I was being shamed for doing something wrong’* (C12, MH Nurse).

When talking about the process of knowledge and skill development, a number of clinicians also argued that supervision is ‘*not necessarily being told what to do*’ but rather providing room to co-construct ‘*creative solutions*’ (C9, Dietitian) and *‘encouraging you to think of the solutions for yourself which improve your development, and then providing feedback on that’* (C5, Dietitian) and a two way process of *‘shared learning’* (C6, Social Worker). These clinician positions emphasised the relational aspect of knowledge acquisition. One supervisor (C19, Clinical Psychologist) positioned reflective supervision as inviting clinicians to reflect on their own practice and in doing so, generate solutions. Another clinician (C25, Psychologist) talked about the developmental trajectory of clinicians and the importance of the opportunity for ‘*more directive*’ supervision for early career clinicians with a greater focus on reflective practice as clinicians gain expertise in the field of eating disorders.

#### Subtheme 2.2: support and reflective practice

Supervision was also talked about as a place that clinicians felt supported, including space to debrief. Topics clinicians experienced as helpful to de-brief included struggles associated with client distress and safety, treatment system constraints, and workplace conflict and pressures (for example, working with individuals whose care needs to be escalated but did not meet criteria for hospital admission). Supervision was also perceived as helpful when addressing clinician anxiety (and other emotions) in working with people who were distressed in the context of living with an ED. In their interviews, clinicians highlighted a ‘*parallel process*’ (C8, Dietitian) of anxiety where they noticed how they had picked up on an individual’s (or families) distress and found themselves for example, awake at *‘three o’clock in the morning’* (C3, Dietitian) or *‘holding a lot of risk’* (C18, Psychologist). Supervision was described by some clinicians as a place that they could feel reassured that they had done everything and the impacts of this was depicted by C7 (Dietitian) who said that: ‘*You know you ticked all the boxes. Then you can kind of sleep well at night if it’s been a difficult case.’*

Another role of supervision in preventing clinician burnout and supporting ethical clinical practice was in the marking out of boundaries between the personal and the professional and in supporting clinicians to regulate their own responses, including countertransference, to individuals (and families) they treat. Clinicians across the professions talked about the importance of supervision as a place where they could examine and mark out boundaries in the therapeutic relationship *(‘boundary drift’*—C9, Dietitian) and in the context of workplace practices (C8, Dietitian). Some of the clinicians also talked about supervision as an important process in them maintaining professional boundaries in the context of work that at times was challenging. Clinicians reflected on how these conversations could be challenging (‘*tricky*’- C25, Psychologist; ‘*hard*’-C24, Psychiatrist) but ‘*necessary*’ (C24) in navigating boundaries in clinical contexts that were perceived as ‘*grey*’ (C25) and ‘*blurry*’ (C3, Dietitian).

Many of the interviewed clinicians talked about experiences of reflective practice in supervision from a range of different perspectives. Outside of the assumption that reflective practice is inviting the clinician to reflect on their own work to generate solutions, other experiences of reflective practice included: (a) unpacking assumptions and stretching perspectives; and (b) facilitating the strengthening of a clinician’s sense of professional identity. For example, the role of supervision in inviting clinicians to unpack their taken-for-granted assumptions was evident in clinician’s accounts of ‘*shedding that mindset … every [therapy] session has to be productive’* (C8, Dietitian) and identifying ‘*blind spot(s)*’ (C18, Psychologist). For the other participants, reflective practice facilitated greater ‘*awareness*’ (C13, MH nurse) of what was there but ‘*you’re missing*’ (C18, Psychologist) and generating ‘*new ways*’ of working that ‘*resonate*’ (C3, Dietitian) with people with whom they worked. In doing so, reflective practice also provided scope for clinicians to sharpen what Hewson and Carroll [34] term their ‘practice framework’ that had implications for their therapeutic work beyond a singular context. Reflective practice was also experienced by a number of clinicians as helpful in the negotiation and strengthening of their identities. For example:

*EXTRACT 4*

*… if you don’t have that protected space for reflection and for debriefing, it’s really hard to get a grip on your identity as a clinician, and therefore that potentially impacts how you work with people, and particularly in the eating disorder space, I feel like being genuine and being authentic for the long-term care is really, really important. (C10, Dietitian)*

Addressing the person of the clinician in supervision was experienced by this clinician as a part of being ‘*genuine*’ and ‘*authentic*’ (C10, Dietitian) in their work. Relatedly, the acknowledgement that the person with a lived experience is inevitably ‘*impacted by your own personhood*’ (C25, Psychologist) that is, the person of the therapist, was also raised.

Supervision was also talked about as a space to process clinicians’ experiences as they witnessed traumatic accounts of individual’s lived experiences (C4, Dietitian). This essential role of supervision in supporting clinicians in preventing ‘*burnout*’ was talked about by a number of clinicians, regardless of their clinical experience. Implicit in their accounts was a view of supervision as a place to preserve a sense of professional identity in the context of challenges that they faced in their day-to-day work.

#### Subtheme 2.3: supervisory context

Twenty-six (93%) clinicians had experienced individual supervision, and the supervision experiences outlined above were mainly spoken about in the context of individual supervision. Most clinicians (n = 24; 86%) reported experiences of supervision in group contexts, with around half of these experiencing peer supervision (n = 13; 46.4%) and around half of the clinicians (n = 15; 53.6%) had experiences of supervision in an interdisciplinary context (see Table 2).

Clinicians who experienced group supervision cited strengths of this format as widening their perspectives on their practice through witnessing the multiple perspectives of group members. For example, this was described as ‘*more brains being in the room*’ (C27, Social Worker), ‘*another way of looking at things*’ and ‘*a different lens*’ (C24, Psychiatrist). On the other hand, clinicians also described some challenges in group supervision including less opportunities to find a safe space to engage in more detailed conversations that involved support, debriefing, and reflective practice opportunities (theme 2.2). Further, clinicians from some professions experienced greater challenges in feeling safe to have a voice in these group contexts finding it ‘*intimidating*’ (C11, Dietitian), often due to the invisible professional hierarchy that privileges the voice of some professions over others in matters related to EDs and their treatment.

Group experiences that were described where clinicians felt safe and supported to talk about their clinical work included one ‘*supervision group*’ with the same membership and regular meetings. This clinician experienced group supervision as an opportunity to ‘*build a safe space*’ (C18, Psychologist) for presenting and collaborating in their work. Another clinician talked about how witnessing other clinicians struggling in their work reduced the sense of isolation in their work ‘*I could see everyone else in my group was struggling just as much as I was*’ (C25, Psychologist). Implicit in this clinician’s experience is the potential for group supervision to provide a safe space to ameliorate a sense of isolation and build a community where witnessing others’ work has scope to build and preserve a clinician’s sense of professional identity.

### Cross-cutting theme 1: clinician experience & practice

Clinicians’ level of experience and years of practice influenced their attitudes towards the supervision and training requirements to maintain their credentialing. While most early career clinicians valued the need for ongoing supervision and other CPD activities, experienced clinicians tended to have more conflicting attitudes with some believing the requirements are reasonable and others suggesting a more tailored approach based on level of experience was necessary.

In setting up the supervision space, early career clinicians tended to prioritise a supervisor’s expertise as an ED clinician. Although the credential was a guide for clinicians in ascertaining a supervisor’s level of experience, this was not viewed by all clinicians as mandatory in their choice of supervisor. More experienced clinicians tended to emphasise less about the clinicians’ experience in the field of EDs and more about the supervisor’s experience and expertise in supervisory practices, including reflective practice.

Regardless of the experience and practice in the field of EDs, the clinicians were seeking a goodness of fit in supervision that was:

1. Tailored to their needs, preferences, and developmental stage as clinicians (like learning how to cook); and
2. Experienced as a safe space to scaffold their knowledge into clinical competency, debrief and process anxieties that arose in their work, and to build their identities as clinicians, including through reflective practice. Some clinicians reflected on the parallel process of these aspects of the supervisory relationship with key ingredients of the therapeutic relationship.

### Cross-cutting theme 2: enablers and barriers

Clinicians identified a number of enablers and barriers in establishing goodness of fit supervision and in engaging with other CPD activities. Barriers that were evidenced across the main themes included time, cost, lack of support from workplace, and access to CPD activities and supervisors who were experienced in the treatment of EDs and the practice of supervision itself (including in rural and remote areas in Australia). Enablers for CPD, including supervision, was finding a supervisor through the NEDC CPD programs, engagement in multidisciplinary supervision groups, and flexibility for online learning and supervision.

## Discussion

The present study’s thematic analysis synthesised the perceptions and experiences of CPD requirements for credentialed clinicians following the recent introduction of the ANZAED Eating Disorder Credential. Two main themes clustered around clinicians’ individual CPD priorities and their subsequent CPD experience. The study found that clinicians reported their preferences when setting up their CPD supervision space, highlighting the importance of a supervisor with expertise in both EDs and the practice of supervision itself and the profound need for safety in the supervisory relationship. However, it was evident that some clinicians expressed concern about accessing a supervisor who was also a Credentialed Eating Disorder Clinician, unless they had been recipients of the NEDC package, and may not have been aware that supervisors are only required to be eligible for the Credential. When reflecting on the ongoing requirements for maintaining the Credential, more experienced clinicians emphasised the need for flexibility and suggested that CPD requirements needed to be scaffolded to the clinicians’ needs and level of experience.

Within the supervision space itself, credentialed clinicians perceived ED supervision as a key place to for them to build skills, expertise, and confidence in the tailoring of evidence-based ED interventions to the unique needs and preferences of those living with EDs and their families. This included specifically addressing clinical concerns specific to EDs, including physical health care status and safety. Some clinicians also talked about experiences of supervision that went beyond the implementation of specific evidence-based ED interventions to therapeutic interventions that addressed the unique and complex challenges faced by those living with EDs. Furthermore, some of the clinicians, particularly those more experienced, described supervision as more than the implementation of evidence-based ED interventions for individuals and families. These clinicians talked about the imperative of supervision to provide opportunities for reflective practice that enabled (re)consideration of their practice frameworks and the further development of the person of the clinician. This was positioned by many clinicians as imperative to the development of the clinician including in prevention of clinician burnout. These roles of supervision in these clinicians’ experiences align with Sackett’s well-known emphasis on the arm of clinician expertise in the delivery of evidence-based practice. 

Clinicians, particularly those who were early in their careers, valued supervisor experience and the opportunity to increase their knowledge and competency. Clinicians also perceived CPD supervision as a place of two-way learning where their knowledge, skills, and abilities were understood and respected by their supervisors and supervision was about inviting, encouraging, and empowering clinicians to explore what they didn’t know they already knew. These diverse perspectives highlighted the need for supervision to be tailored to the clinician’s level of experience and expertise both broadly and in working with EDs. These findings support Boie & Lopez’s [1] application of the ‘Integrated Developmental Model (IDM)’ [35] to ED supervision as a framework to facilitate supervisees’ growth as they move through the levels of professional development. In this model, supervisors tailor supervision to the needs of supervisees according to proposed structures of motivation, autonomy, and self-other awareness across the eight domains of clinical practice (e.g., client conceptualization, treatment plans and goals, and professional ethics, etc.). Boie and Lopez [1] found that whilst all counsellors will move through the various levels and domains of professional development, there are unique competencies required for counsellors working in the field of EDs including an understanding of countertransference, crisis management, and knowledge of the aetiology of EDs.

For many clinicians, supervision took on more of a case consultation format, serving as the bridge between research and its real-world application and focusing substantially on model fidelity and the avoidance of therapist drift. However, the inherent risks of this more instructional approach to supervision should be noted. Namely, some clinicians perceived feeling disempowered through being encouraged to strictly adhere to evidence-based therapies rather than considering the unique needs of each client, and thus other supervisory components such as reflective practice may be sidelined. While case consultation may touch the surface of a practitioner’s practice framework, reflective practice aims to unearth practitioner assumptions and invite a re-positioning on these [34]. This is designed to transform practitioners’ learning so that it may be applied across a range of individual presentations through the development of insights. The clinicians for whom supervision was experienced as a safe place to reflect on and stretch their assumptions, talked about these experiences in ways consistent to what Hewson and Carroll [34] refer to as ‘an experiential learning space’ where ‘an experience is reflected upon to identify and fine-tune the assumptions that underpin it (the practice framework)’ and ‘the primary goal is discovery—to allow our practice to speak to us’. In the present study, clinicians ascribed a range of meanings to reflective practice. However, supervision as a place to reflect on and unpack assumptions, debrief, process anxieties, and stretch their perspective on their work and themselves as clinicians were perceived to be key components. Unsurprisingly, most clinicians noted feeling more comfortable engaging in reflective practice in an individual supervision format where safety had been established and where there was more time and focus afforded to them. In peer supervision contexts, group supervision was favoured. Notable was the opportunity the clinicians experienced to expand their knowledge and perspectives through both having group members or colleagues with varying levels of experience, and when group members came from other disciplines. Another key element of the supervision space that was prioritised by clinicians, regardless of level of experience, was the ability to ‘*sleep well at night*’ (C7, Dietitian). This recurrent theme highlighted by clinicians demonstrates the responsibility clinicians and their supervisors face when working with people living with the effects of EDs on their physical and psychological wellbeing. Supervision was perceived as serving a safe and non-judgmental space that provided clinicians with another layer of support to mark out the boundaries of the therapeutic relationship and in the prevention of burnout, consistent with previous research on clinical supervision in health care contexts [36–40].

When reflecting on the other ongoing professional development relevant to EDs, the clinicians highlighted the need for requirements to be made clearer and to consider the individual circumstances of clinicians. These included clinical experience, periods of leave from the workforce, and ED client workload and to consider how to make training more accessible and affordable to ensure that the ongoing requirements are realistic and achievable for all clinicians.

This study raised several important considerations for the successful implementation of the Credential and its CPD. These considerations have specific implications for credentialing of clinicians in the ED field (for example, physical health care status and safety in ED contexts) and also have implications beyond the ED field as they may reflect broader issues and concerns of clinician credentialing. First, clinicians would value enhanced flexibility in the Credential to account for the fact that different clinicians will need different things, at different times. CPD requirements ideally would be both scaffolded to a clinician’s level of experience and there needs to be greater clarity in the Credential CPD requirements to stipulate that these include supervision and other CPD activities in ED co-morbidities such as trauma, substance use, and suicidality. The study suggests that supervision may be able to go beyond the case consultation format and to be more inclusive of reflective practice so that clinicians can explore practice frameworks and their practice more broadly and in the context of their unique client presentations. Second, there is a need for the Credential to support the development of the supervisor workforce and improve access to suitable supervisors. Supervisor training is imperative and greater clarity is also needed so that credentialed clinicians are clear that this is also included in the Credential’s CPD activities. Third, a searchable directory of clinical supervisors would improve access for clinicians to experienced supervisors.

Other barriers of time and cost could be addressed by ensuring that there is consideration to how the ongoing CPD requirements can be more closely aligned with the various interdisciplinary professional body requirements (as outlined in Additional File 1). Greater harmonization is likely to help with cost and time efficiencies, particularly for early career clinicians who may be trying to develop their skills across a broader range of areas. Cost could also be subsidized for those people who are returning to the workforce, working part-time, or at a reduced rate. Finally, more experienced clinicians have expressed concerns that there may be a perception that early career clinicians’ expertise is conflated with that of more experienced clinicians and potentially poses a concern for care of clients with more complex presentations. This is despite the current requirements for a baseline level of experience.

The strength of the study included the utlisation of two researchers (JC & MM) who extracted data and agreed on themes. There was also an adequate to large sample and saturation of themes was reached. Another notable strength of the study was that it triangulated the perspectives of Credentialed Eating Disorder Clinicians from multiple professions. However, a limitation was the low representation of some professions including psychiatrists, occupational therapists, and mental health nurses meaning that the data presented largely represents the views of credentialed psychologists and dietitians, who also largely make up the population of Credentialed Eating Disorder Clinicians. It is also acknowledged that the research was conducted in the early stages of the Credential's implementation. While this is beneficial in that it can inform the later stages of the Credential, it is also possible that perceptions were influenced by experiences prior to and in the initial phases of the Credential and are likely to change over time. In addition, it is important to consider that it is possible that clinicians with strong opinions of the Credential were more likely to participate in the research. Nevertheless, the findings of this study need to be understood in light of its qualitative design to analyse the richly textured nature of the experiences of ED clinicians who have sought to be credentialed, rather than to generalise these experiences to all credentialed ED clinicians in Australia.

Further research is needed once the Credential is more established, and this follow up research should aim to provide a more in-depth focus on the experiences of professions that had a low representation in this sample. It is also important to acknowledge that future research is needed to understand the clinical effectiveness of the credential with respect to therapist performance or response of patients undergoing treatment by a credentialed clinician. For example, studies to investigate whether credentialed clinicians are more likely to deliver evidence-based treatments, including appropriate tailoring to the needs and preferences of those with a lived experience. In conclusion, this study found that Credentialed Eating Disorder Clinicians experienced ongoing professional development, including supervision and other CPD experiences, as beneficial in scaffolding knowledge into clinical practice in EDs, and to support them as clinicians, including in the prevention of burnout. However, the requirements of CPD for the Credential were perceived by many as burdensome and/or challenging to meet. This led to preferences for greater flexibility in the CPD requirements and scope to scaffold these to a clinician’s developmental needs, and professional circumstances. Further consideration of how to ensure that the ongoing CPD requirements of the Credential are both valuable and sustainable for all clinicians, regardless of level of experience, is recommended.

## Supplementary Information

Additional file 1.

Additional file 2.

Additional file 3.

Additional file 4.

## Data Availability

The datasets used and/or analysed during the current study are not publicly available. They may be available from the corresponding author upon reasonable request and in accordance with Human Research Ethics permissions.

## Abbreviations

ANZAED: Australian & New Zealand Academy for Eating Disorders
CPD: Continuing professional development
DBT: Dialectical behavior therapy
ED: Eating disorder
ED-EBI: Eating disorder evidence based interventions
IDM: Integrated developmental model
MH: Mental health
NEDC: National eating disorder collaboration
QuEDS: Queensland eating disorder service
