# Supplementary material for: ANZAED eating disorder credentialed clinician perceptions and experiences of professional development
Source: J Eat Disord. 2025 Jul 16;13(Suppl 1):142. doi: 10.1186/s40337-025-01307-w (PMC12265107; doi:10.1186/s40337-025-01307-w)
Supplement: Supplementary file 1 — Additional file 1. [file 40337_2025_1307_MOESM1_ESM.pdf]

ADDITIONAL FILE 1

CPD requirements for ANZAED credential and professions eligible for the credential in Australia

Health Professional	Professional body	Supervision requirements	Other CPD requirements
Dietitians & mental health professionals	ANZAED	Minimum of 6 hours of ED specific supervision per year (at least 3 hours of individual supervision).<br><br>Supervisors must be credentialed or meet the eligibility criteria for the credential [1].	Minimum of 15 hours of other ED specific CPD (e.g., workshops & training, higher education, conferences) [1]
Dietitians	Dietitians Australia	**No supervision requirements**. Provisional accredited practising dietitian (APD) must complete 12 months of formal mentoring.<br><br>Formal mentoring for provisional APD's must be with a full APD [2].	Minimum of **30 hours** of other CPD each year [2].
Counsellors & Psychotherapists	Psychotherapy and Counselling Federation of Australia (PACFA)	Minimum of **10 hours** of formal supervision each year. If a practitioner has accrued over 400 hours of client practice hours per annum, they must undertake 15 hours of formal supervision. Additional hours of supervision are strongly recommended for those with a full practice. For those seeking more senior clinical registration, 75 post-graduation supervision hours with a minimum of 50% as individual supervision. Peer supervision is only for senior practitioners who have been members with PACFA for 5 continuous years and can only make up 50% of the required hours.<br><br>Supervisors must have a minimum of 5 years clinical experience and have supervision competencies equivalent to the requirements of the PACFA supervision training standards. The supervisor does not have to be a PACFA registrant and may be from a cognate field [3].	Minimum of **20 hours** of other CPD annually [3].
	Australian Counselling Association (ACA)	To maintain membership with the ACA, members need to complete a minimum of 10 hours of supervision annually. The ratio of supervision hours should be 1 hour of supervision for every 20 hours of client contact or at least once a week if in full time employment.<br><br>A supervisor needs to be an ACA registered supervisor or an ACA recognised supervisor [4].	Members are required to complete 25 points of professional development per membership year (point allocation for each eligible activity is defined by the ACA) [4].
Mental health nurse	Australian College of Mental Health Nurses (ACMHN)	No specific supervision requirements.	A **minimum of 50 hours** of CPD over the previous 12 months (40 hours must be relevant to mental health

			nursing and up to 10 hours does not need to be directly related to mental health)<br><br>CPD is categorised into two distinct domains:<br>Education related activities (professional education; minimum of 20 hours)<br>Practice related activities (practice development; minimum of 20 hours) [5].
Nurse practitioners	Australian Health Practitioner Regulation Agency (AHPRA)	Supervision is a requirement for nurse practitioners with a registration requirement, notation, condition, or if there is an undertaking recorded on the practitioner's registration [6].	**Minimum of 30 hours** of CPD (20 hours for registered nurse + 10 hours relating to prescribing and administering medication, diagnostic investigations, consultations, and referral) [7].
Occupational therapists	Australian Health Practitioner Regulation Agency (AHPRA)	Supervision is a requirement for practitioners with limited, provisional registration, or conditions on their registration. Supervision requirements will vary for each practitioner.<br><br>Supervisors should have their general registration and have at least two years of experience [8].	A **minimum of 20 hours** of CPD yearly, including a minimum of 5 hours of CPD in an interactive setting with other practitioners [9].
Psychiatrists	Australian Health Practitioner Regulation Agency (AHPRA)	Psychiatrists are required to participate in practice peer review. However, there are no specific formal supervision requirements [10].	Psychiatrists need to meet the requirements of a CPD program of an accredited CPD home. A **minimum of 50 hours** of CPD activities each year that are made up of the following:<br>• At least 12.5 hours in educational activities.<br>• At least 25 hours in activities focused on reviewing performance and measuring outcomes.<br>The remaining 12.5 hours across any CPD type [11].
Psychologists	Australian Health Practitioner Regulation Agency (AHPRA)	Psychologists must complete **10 hours** of peer consultation annually. This includes supervision, mentoring, and consultation in a one-on-one or group format.<br><br>Supervisors should be more experienced and knowledgeable than the supervisee [12].	Psychologists need to complete **20 hours** of other CPD activities annually[12].
Social workers	Australian Association of Social Workers (AASW)	Social workers must complete a **minimum of 10 hours** of supervision, professional mentoring, professional coaching and professional consultation.<br><br>It is preferred that supervision is conducted by a social worker, but it isn't a requirement [13].	Accredited social workers need to complete a **minimum of 30 CPD hours** annually to maintain their accreditation. CPD is divided into categories:<br>1. Skills and knowledge (minimum 15 hours)<br>2. Professional identity (minimum 5 hours)<br>3. Supervision (10 hours)

			Any accredited mental health social workers also need to complete an additional 10 hours of CPD that is on focussed psychological strategies [13].

References

1. Australian and New Zealand Academy of Eating Disorders. Connect-ed [Internet]. Castlecrag (AU): Australian and New Zealand Academy of Eating Disorders; 2024 [cited 2025 May 18]. Available from https://connected.anzaed.org.au/
2. Dietitians Australia. Working in nutrition and dietetics in Australia: Apply to become and Accredited Practicing Dietitian [Internet]. Woden (AU): Dietitians Australia; 2025 [cited 2025 July 9]. Available from: https://dietitiansaustralia.org.au/working-dietetics/credentialing-dietitians
3. Psychotherapy and Counselling Federation of Australia: CPD and supervision standards [Internet]. Carlton (AU): Psychotherapy and Counselling Federation of Australia; 2024 [cited 2025 July 9]. Available from: https://pacfa.org.au/portal/Membership/CPDSupervision.aspx
4. Australian Counselling Association: Register with ACA: Annual registration renewal requirements [Internet]. Newmarket (AU): Australian Counselling Association; 2025 [cited 2025 July 9]. Available from: https://theaca.net.au/registration/registration-categories
5. Australian College of Mental Health Nurses. Credential for Practice Program Evidence Based Record of CPE and CPD. 2020 [cited 2025 July 9]. Available from: https://acmhn.org/common/Uploaded%20files/PDFs/Recredentialing/ACMHN_EBR_Guide_20Feb2020.pdf
6. Nursing and Midwifery Board AHPRA. Supervised Practice [Internet]. Australia: AHPRA; 2022 [cited 2025 July 9]. Available from: https://www.nursingmidwiferyboard.gov.au/Registration-and-Endorsement/Supervised-practice.aspx
7. Nursing and Midwifery Board AHPRA. Fact sheet: Continuing Professional Development [Internet]. Australia: AHPRA ; 2025 [cited 2025 July 9]. Available from: https://www.nursingmidwiferyboard.gov.au/codes-guidelines-statements/faq/cpd-faq-for-nurses-and-midwives.aspx
8. AHPRA. Supervision Guidelines [Internet]. Australia: AHPRA; 2025 [cited 2025 July 9]. Available from: https://www.ahpra.gov.au/Resources/Supervised-practice/Supervision-guidelines
9. Occupational Therapy Board of Australia. Guidelines: Continuing Professional Development. 2019 [cited 2025 July 9]. Available from: https://www.occupationaltherapyboard.gov.au/documents/default
10. Medical Board AHPRA. Professional development plan and types of CPD [Internet]. Australia: Medical Board of AHPRA; 2024 [cited 2025 July 9]. Available from: https://www.medicalboard.gov.au/Professional-Performance-Framework/CPD/Professional-Development-Plans
11. Medical Board AHPRA. What do I need to do? Professional performance framework [Internet]. Australia: Medical Board of AHPRA; 2024 [cited 2025 July 9]. Available from: https://www.medicalboard.gov.au/Professional-Performance-Framework/CPD/What--do-I-need-to-do
12. Psychology Board AHPRA. Continuining professional development [Internet]. Australia: Psychology Board AHPRA; 2019 [cited 2025 July 9]. Available from: https://www.psychologyboard.gov.au/Registration/Continuing-Professional-Development.aspx
13. Australian Assocation of Social Workers. Continuing Professional Development Policy. 2025 [cited 2025 July 9]. Available from: https://aasw-prod.s3.ap-southeast-2.amazonaws.com/wp-content/uploads/2023/06/aasw-cpd-policy.pdf
