# Supplementary material for: ANZAED eating disorder credentialed clinician perceptions and experiences of professional development
Source: J Eat Disord. 2025 Jul 16;13(Suppl 1):142. doi: 10.1186/s40337-025-01307-w (PMC12265107; doi:10.1186/s40337-025-01307-w)
Supplement: Supplementary file 2 — Additional file 2. [file 40337_2025_1307_MOESM2_ESM.pdf]

ADDITIONAL FILE 2

Comparing the Clinician Characteristics of Participants who Completed the Survey and Interview, Participants who Completed the Survey Only, and Participants who Completed the Survey Only but Indicated Interest to be Interviewed.

Clinician characteristics	Survey & Interview (n = 28)	Survey only (n = 200)	Survey only but indicated interest to be interviewed[7] (n=29)	Statistical testing (n =57; 28 vs 29)	Statistical testing (n =228; 28 vs 200)
	**Mean ± SD**			**T-test (p)**	
Age (years)	41.9 (13.9)	37.8 (9.3)	38.6 (8.76)	-1.06 (.290)	-2.036 (.043)
Clinician years of experience	12.6 (10.7)	11.2 (7.7)	11.8 (7.3)	-.313 (.756)	-.887 (.376)
	**n (%)**			**χ^2, df (p)**	
Years of ED specific experience				.017, 1 (.896)	.121, 1 (.728)
<5 years of experience	14 (50%)	93 (46.5%)	14 (48.3%)		

5+ years of experience	14 (50%)	107 (53.5%)	15 (51.7%)		
Identified gender				.393, 2 (.822)[8]	1.323, 2 (.516)[8]
Female[1]	25 (89.3%)	186 (93%)	27 (93.1%)		
Profession				4.362, 2 (.113)	3.795, 2 (.150)
Psychologist	9 (32.1%)	86 (43%)	17 (58.6%)		
Dietitian	10 (35.7%)	80 (40%)	5 (17.2%)		
Other mental health professional[2]	9 (32.1%)	34 (17%)	7 (24.1%)		
Most common clinical area of work[3]				9.988, 1 (.002)	4.944, 1 (.026)
Anorexia Nervosa & Atypical Anorexia Nervosa	23 (82.1%)	121 (60.5%)	12 (41.4%)		
Other	5 (17.9%)	79 (39.5%)	17 (58.6%)		
Practice setting				.197, 2 (.906)	4.709, 2 (.095)
Private	11 (39.3%)	109 (54.5%)	13 (44.8%)		
Public	6 (21.4%)	49 (24.5%)	6 (20.7%)		
Private & public	11 (39.3%)	42 (21%)	10 (34.5%)		

Location of practice				.436, 1 (.509)	.000, 1 (.982)
Metropolitan	18 (64.3%)	129 (64.5%)	21 (72.4%)		
Regional, rural, remote, or mixed	10 (35.8%)	71 (35.5%)	8 (27.6%)		
Treatment format[4]				.969, 2 (.616)[8]	2.785, 2 (.248)[8]
In person	4 (14.3%)	49 (24.5%)[5]	7 (24.1%)		
Telehealth	4 (14.3%)	14 (7%)[5]	3 (10.3%)		
Multiple formats	20 (71.4%)	136 (68%)[5]	19 (65.5%)		
Hours/week of ED work				7.249, 1 (.007)	1.802, 1 (.179)
<16 hours a week	15 (53.6%)	133 (66.5%)	25 (86.2%)		
16+ hours a week	13 (46.4%)	67 (33.5%)	4 (13.8%)		
Proportion of clients with an ED				4.341, 3 (.227)[8]	4.350, 3 (.226)
A few (<25%)	4 (14.3%)	52 (26%)	11 (37.9%)		
A substantial number (25-50%)	9 (32.1%)	44 (22%)	8 (27.6%)		
Many (50-75%)	4 (14.3%)	47 (23.5%)	3 (10.3%)		
Almost all/All (75-100%)	11 (39.3%)	57 (28.5%)	7 (24.1%)		

Provides clinical supervision[4,5]	10 (35.7%)	79 (39.7)	11 (37.9%)	.183, 2 (.913)[8]	1.704, 2 (.427)[8]
NEDC package recipient[6]	13 (46.4%)	N/A	N/A		

[1] Cell counts for other genders suppressed to protect confidentiality of sensitive/identifying information.

[2] Other mental health professionals included counsellors, mental health nurses, social workers, and a psychiatrist.

[3] These are exclusive categories where participants ranked their most commonly seen client diagnosis and data were grouped for the purposes of statistical analysis. Other included Bulimia Nervosa, Binge Eating Disorder, Subthreshold Bulimia Nervosa, Subthreshold Binge Eating Disorder, Purging, Night eating, Avoidant Restrictive Food Intake Disorder and Other.

[4] This analysis was missing data, n = 227.

[5]Includes those who were both a supervisor and a supervisee. Three groups were compared supervisee only, supervisor and both supervisor and supervisee.

[6] This data was derived from the interviews and was not asked in the survey.

[7]These 29 participants indicated an interest to be interviewed but were not (either declined, were ineligible, or interviews had reached data sufficiency). Participants who were not interviewed saw a smaller proportion of clients presenting with Anorexia Nervosa (AN) or Atypical AN (p = .002) and worked less hours a week with eating disorder clients than those participants who were interviewed (n=28).

[8] Fisher’s Exact test used for cell sizes less than n=5.
