# Supplementary material for: ANZAED eating disorder credentialed clinician perceptions and experiences of professional development
Source: J Eat Disord. 2025 Jul 16;13(Suppl 1):142. doi: 10.1186/s40337-025-01307-w (PMC12265107; doi:10.1186/s40337-025-01307-w)
Supplement: Supplementary file 3 — Additional file 3. [file 40337_2025_1307_MOESM3_ESM.pdf]

# ADDITIONAL FILE 3

## Clinician Semi Structured Interview Questions

| **Questions for all Clinicians** | |
|---|---|
| *Questions* | *Further Prompts* |
| 1. What is your profession? | o How long have you been doing this work for?<br>o Do you work primarily with people with ED? |
| 2. What are your views in general on a credentialing of clinicians? (what is good / not good about it in your opinion?) | o What does the existence of a Credential that recognises the expertise and training of clinicians mean to you? |
| 3. How did you first hear about the ANZAED Eating Disorder Credential? | o What does the existence of a Credential that recognises the expertise and training of clinicians mean to you?<br>o How long have you been credentialed? |
| 4. What motivated you to become an ANZAED Eating Disorder Clinician? | o What aspects of the Credential did you find appealing?<br>o Was there anything that made you second guess if you should become credentialed?<br>o Were there any significant barriers to attaining the Credential? |
| 5. Since becoming credentialed, what has been good/not good about it? | |
| 6. How does it make you feel to be credentialed? How do you see yourself? | o Has being credentialed impacted upon your clinical work (e.g. referrals, quality of work, capacity to do work) |
| 7. Are you aiming to maintain your credentialing status into the future? Why/why not? | o Do you think the ongoing professional requirements for maintaining your Credential status are reasonable? Why/why not? |
| 8. Have you ever used the Connect•ed website? | o How did you find the process of setting up your clinician profile?<br>o Have you used the Find a Treatment Provider Directory for patient referral?<br>o What was good/not so good about the directory? Anything you would change about it? |
| 9. Since becoming credentialed, have you provided ED supervision, participated in supervision for EDs, or both? | |

| **Questions for Supervisees** | |
|---|---|
| *Questions* | *Further Prompts* |
| 10. How have you found accessing supervision for ED work? | |
| 11. What are some of the things you are looking for in a supervisor? | ○ What are the skills that you think an ED supervisor should have? What does a good supervisor look like? |
| 12. Since becoming credentialed, can you tell me about your ED supervision experiences? | ○ Current supervisor (Profession, length of time, frequency, type)<br>○ Has supervision impacted your clinical work with EDs?<br>○ What has been helpful/useful about supervision?<br>○ Any difficulties in supervision? Tell me about it? How does this impact you? What does this mean for you as a clinician?<br>○ Have there been topics that were easier or more difficult to broach and discuss with your supervisor?<br>○ Anything else you need or would like from supervision? |
| 13. What aspects of supervision have you found to be essential in your ongoing development as an eating disorder treatment provider? | ○ Do you feel that supervision has improved your confidence / competence / ethical decision making / reflective capacity? If yes, in what way? If not, why not?<br>○ What could have been done differently? |
| 14. Have you received supervision from a professional outside of your own profession? | ○ What was good / not good about it?<br>○ Were there any additional challenges within/across disciplines?<br>○ If you have not received supervision outside of your profession, would you consider this? Why/why not? |
| 15. Have you participated in other forms of supervision such as peer and/or group? What was that like? | ○ Good/not so good compared to individual supervision? |

| **Questions for Supervisors** | |
|---|---|
| *Questions* | *Further Prompts* |
| 16. Since becoming credentialed, can you tell me about your experience of supervising clinicians for ED specific supervision? | ○ How long have you been providing supervision? How many supervisees? |
| 17. What are the benefits/challenges, do you think, in providing ED specific supervision? | ○ Valued? Supervision prioritised by your company/organisation? Time allocated to provide it?<br>○ How well equipped do you feel to provide ED specific supervision? |

| | |
|---|---|
| | ○ What contributes to feeling well/not well equipped? |
| 18. Have you provided supervision for a professional outside of your own profession? | ○ Can you tell me about that?<br>○ How did you find it/feel about it?<br>○ Did you feel competent?<br>○ If not, would you consider this? Why/ why not? |
| 19. Have you found credentialed helpful/not helpful as a supervisor? | ○ Have you encouraged your supervisees to become credentialed? Why/why not? |

| **Questions if not an ED Supervisor** | |
|---|---|
| *Questions* | *Further Prompts* |
| 20. If you are not an ED supervisor, why not? Would you consider it in the future? What would you need to do? | |

| **Other Questions** | |
|---|---|
| *Questions* | *Further Prompts* |
| 21. In your view, is there anything else that organisations such as ANZAED or other professional organisations can be doing to support eating disorder specific supervision? | ○ Do you think that receiving training in eating disorder specific supervision will assist you in your supervisory role? Why or why not?<br>○ Do you think that being provided a set of guidelines specific to the supervision of eating disorder treatment will assist you in providing supervision? Why or why not? |
| 22. Is there anything else that we have not covered today that you think might be important to mention? | |
| 23. During the interview, what stood out for you today? What was been most helpful / not helpful today? Why? | |
| 24. What is the take home message you want us to have? | |

## End of interview
