# Supplementary material for: ANZAED eating disorder credentialed clinician perceptions and experiences of professional development
Source: J Eat Disord. 2025 Jul 16;13(Suppl 1):142. doi: 10.1186/s40337-025-01307-w (PMC12265107; doi:10.1186/s40337-025-01307-w)
Supplement: Supplementary file 4 — Additional file 4. [file 40337_2025_1307_MOESM4_ESM.pdf]

ADDITIONAL FILE 4

Exemplar Data Extracts for Themes Identified from Clinicians Semi-Structured Interviews

Themes	Sub-Themes	Exemplar Extracts
Theme 1: Clinician CPD priorities	Subtheme 1.1 Supervisor expertise and competence	C4: *"I suppose we were looking for someone who had been in the area for a number of years. There wasn't a set timeframe, but I suppose, like the expertise that comes with that. And so, the person that we found has been working in eating disorders for twenty to thirty years, and has a number of publications behind him, and his practice and his principles are very much in line with what our hospital does"* (Dietitian)<br><br>C18: *"But I do want someone who has a lot of experience in the field. It helps me to feel that I'm getting the supervision that I need. I think, for where I'm at in my career I want someone with experience with eating disorders and other quite significant comorbidities as well."* (Psychologist)<br><br>*C7: "Yeah, probably years of expertise. The additional training that they might have done in eating disorders like, you know, motivational interviewing, or CBT, or something like that and then also the number of years that they've been a supervisor. Yeah, that's the most important things."* (Dietitian)
Theme 1: Clinician CPD priorities	Subtheme 1.2 Supervisory relationship	C1: *"[....] I think length of experience is crucial and a personality type that lends itself to being, you know, collaborative, not like: "I'm your teacher, listen to me, this is what you should be doing, that's all wrong, that's rubbish, you need to be doing this"* (Clinical Counsellor*)*<br><br>C14: *"I think everybody, kind of, probably responds differently to different supervisors, and so, I think some approaches work better than others. My individual supervisor was really very nice and affirming, which is nice, but I was like, I kind of want more critiquing, just hearing "all those things sound good" is nice, but not particularly helpful."* (Clinical Psychologist)<br><br>C18: *"Hmm, okay, if I answer it maybe in a couple of different ways. So, on a more personal level I want someone who helps me to feel heard, non-judged. safe and comfortable to bring up any challenges and someone who's willing to be curious*

		and collaborative in providing this supervision in terms of what I'm looking for more from a skill set perspective experience is important. I'm not sure what bias I hold there." (Psychologist)
Theme 1: Clinician CPD priorities	Subtheme 1.3 CPD requirements and access	C4: "*I think initially it [accessing supervision] was challenging. I think working in eating disorders is a very busy area and often understaffed. So we know that through the pandemic our referrals have dramatically blown out across the country, so finding someone who has time to provide supervision was challenging and finding someone with the level of expertise that we were looking for was also challenging. I think it will get easier in the future as more people kind of go through this [credentialing] process. But I think initially, just yeah, finding someone that has time was probably the biggest thing. So, we set up group supervision sessions, just the opportunity to make I suppose the most bang for buck out of the time that we spent with the expert in the area given how precious his time is and all the work that he's doing."* (Dietitian)<br><br>C17: *"Yeah, I do. I think it's [reasonable]. I guess because I'm early career though, I'd be interested to know if that shifted in a few years, whether I would feel differently. I feel like there's so many areas of eating disorders that I haven't been able to, I guess in particular on the treatment side, that I haven't done yet… So, I kind of look at the hours and go, well if I did one of those 2-day trainings a year plus some supervision I'd meet that quite easily. But… I kind of wonder you know, in 5, 10 years' time if I was maintaining this, would I be looking at that kind of going maybe "do I need to be doing that many hours?" or would that naturally happen anyway still? I don't know how much that, based on my current level of experience, versus would I look at that differently down the track."* (Psychologist)<br><br>C2: *Yeah. So, I'd say it's a bit tricky. I've got a number of different organizations that I have to do professional development and supervision for, and this will be about the fourth one. I don't know if I'm going to be able to maintain that because of the cost, and because I have to find a completely different supervisor from the ones I already have."* (Counsellor)<br><br>C7: *It's reasonable for me, because eating disorders is like, as I said, that's at least 50% or more of my clientele. And so, the 15 hours of CPD is 50% of my CPD…You know, perhaps, if you're a dietitian that wasn't seeing as many eating disorder clients, and you were doing other areas of practice as well, you'd probably find it too hard. But then again, it's probably better if eating disorder dietitians are more*

		focused on it because then they're going to be better clinicians. So, I think it's probably reasonable. (Dietitian)
Theme 2: The CPD experience	Subtheme 2.1 Knowledge and competency	C16: *"I think as opposed to the CBT-E training, I learned (in individual supervision) that there [...] are choices and options [...] the structured program will work for some people and that they don't yet know exactly who those people are until you got started. [...] whereas the individual supervisor [...] had a little bit more input on different models, and how different models might fit with different situations and different client presentations [...] that's where the choice started coming into it, because we realized that you know, one size does not fit all [...] and there are different cultural complexities within Australia that aren't necessarily addressed."* (Psychologist)<br><br>C11: *"[...] like dotting your I's and crossing your T's, making sure your plan is sound and appropriate for that person is so important, and I think that's what's helped the most, and knowing that it's safe, and it's a collaborative space that we can discuss those things. And I'm not going to get essentially reprimanded for it, but just adapt a plan before it gets too far down the line."* (Dietitian)<br><br>C5*: "I guess the format my supervisor uses which is part of supervisor training using the CLEAR model, which I guess, rather than the supervisor just telling you what to do. They are encouraging you to think of the solutions for yourself which improve your development, and then providing feedback on that."* (Dietitian)<br><br>C17: *"Yeah, yeah, I think, with eating disorder work, it's been really building up my confidence around assessing and managing eating disorder risk which I think has been, you know, it's really different to other areas because of the physical health component and being sort of across that enough. Because obviously I'm a psychologist, I'm not a doctor. I don't have medical training, but being able to understand those thresholds for how urgently does this need to be responded to, when do I need to say, okay, actually, I think you need to go to emergency versus look I think you need to see your GP in the next few days, or yes, you keep your appointment in a couple of weeks, and that's, you know, probably sufficient. Or being able to even give that Psycho-ed to people like "okay if X Y Z happens, then you need to go to emergency", because I think if I didn't have good supervision around the physical health risks as well as the psychological risks, I don't think the work would have been sustainable for me. I think it's obviously such an important area and people can be, yeah,*

		they can be in real danger physically, with these illnesses. It's such a big responsibility (Psychologist)
Theme 2: The CPD experience	Subtheme 2.2 Support and reflective practice	C22: *"I think for me it's having the safe space to process. Obviously, a lot of things pop up throughout the day and the week, and the month. So, having a safe space to unpack that and to understand that. For me it's more the clinical stuff is helpful in terms of how to manage this specific scenario, but for me the main take-home, the biggest thing is how to manage, how to regulate myself in the room with clients and I feel that if I can tend to myself better than I can tend to them better. That's probably that's been where my biggest learning has come from...because I've definitely been close to burning out in the past between studying and working and all of these things. And so, yeah, it's that to me, feels more pertinent and helpful for the longevity of my career and my mental health than bringing a complex case. To me that's the best use of that hour of our time together and maybe that will change.* (Dietitian)<br><br>C4: *[...] the opportunity to debrief and talk through situations, [...] just to kind of reflect and share, given how traumatic some of these experiences can be [...] I think that's been like super, super important. I think this workload has a very high burnout rate and just being able to just be like "I don't know what I just dealt with today", and that being normal and okay.* (Dietitian)<br><br>C27: *"I think reflective practice is really important to me in supervision. I think context is really important. I would need someone who's able to think about like who I am as a practitioner in addition to like what's happening for me personally and be able to like use that in supervision. I need to be challenged in supervision, but I also need to be able to be honest in supervision."* (Social Worker)
Theme 2: The CPD experience	Subtheme 2.3 Supervisory context	C4: *I think that the group supervision probably doesn't act as like something where you can just sound board, and just say "I've had a really like difficult patient, and I'm just feeling really deflated from it". So, I think it's more, I suppose, more formal process. I think the kind of one-on-one supervision kind of really allows the opportunity to have that deep discussion about my own feelings and emotions which is something that I wouldn't bring up in a group forum so I suppose that's probably a big difference."* (Dietitian)

	C2: *"[…] in the group supervision, just not feeling that I could talk about some things because I was different, as most of the group members were psychologists."* (Counsellor)<br><br>C11: *"The group supervision can sometimes be a bit intimidating because it's like 7 different clinicians on a teams meeting picking apart your clients, and that can be a little bit, a little bit confronting, so I prefer that one on one kind of informal chat."* (Dietitian)<br><br>C27: "*I think I go to all of them for very different reasons. So, I have you know, one supervisor for hospital stuff, one supervisor for private practice stuff, one group supervision for eating disorder professionals, one group supervision for team structure, and then one supervision for the family therapy stuff. So, they all kind of have a really distinct purposes, I think. One-on- one supervision is more like the only person's goals or things to talk about in the session are mine. I don't have to share that space. But then, group supervision, I get more like ideas from more brains being in the room, so um they're equally kind of important and valuable to me which is why I do so many of them.* (Social Worker)
